# Immediate needs and level of anxiety of families with traumatic brain injury patients admitted to ICUs

**DOI:** 10.1186/cc11101

**Published:** 2012-03-20

**Authors:** S Gholamzadeh, R Abdoli, F Shariff, R Gholamzadeh, A Maraghian Mohammadi

**Affiliations:** 1 Shiraz University of Medical Sciences, Shiraz, Iran

## Introduction

Meeting the needs of family members of patients in the ICU is an important criterion in assessment of quality of care in the ICU. Therefore this study was conducted to determine the immediate needs and level of anxiety of families with traumatic brain injury patients admitted to ICUs in Shiraz, Iran in 2008.

## Methods

In this descriptive cross-sectional study, a convenience sample of 60 family members was recruited over a period of 4 months. On the second day of ICU admission, one family member for each patient who met the study criteria were asked to complete three questionnaires, consisting of the Critical Care Family Need Inventory (CCFNI), the Statetrait Anxiety Inventory (STAI) and a demographic data sheet.

## Results

The mean ages of the subjects were 32.2 years. A total of 10 needs statements in the CCFNI were rated to be important or very important needs by 50 of the 60 families (83.3%); seven were needs for assurance, two were needs for information, and one of them was needs for proximity. The mean of CCFNI satisfaction scores were low (16.5 ± 1.5) for needs to comfort, and high for needs to support (38.1 ± 4.7). Also the mean score of state anxiety (56.75 ± 5.7) and trait anxiety score (52 ± 6.2) was higher than previous studies.

## Conclusion

A needs-based education program can decrease the level of family anxiety and increase the level of satisfaction.

